# Comparison of 4 sinus augmentation techniques for implant placement with residual alveolar bone height ≤3 mm

**DOI:** 10.1097/MD.0000000000023180

**Published:** 2020-11-13

**Authors:** Chia-Fang Tsai, Whei-Lin Pan, Yi-Ping Pan, Chiu-Po Chan, Yuh-Ren Ju, Yuan-Min Wang, Cho-Ying Lin, Chi-Ching Chang

**Affiliations:** a Department of Periodontics, Chang Gung Memorial Hospital, Taipei; b Graduate Institute of Dental and Craniofacial Science, Chang Gung University, Taoyuan; c Department of Nutrition, Chang Gung Memorial Hospital, Keelung and Chang Gung University, College of Medicine, Keelung, Taiwan.

**Keywords:** atrophic maxilla, BAOSFE, dental implants, lateral window sinus augmentation, transalveolar sinus augmentation

## Abstract

This study compared implant outcomes following maxillary sinus floor augmentation (MSFA) in edentulous patients with a residual alveolar bone height ≤3 mm. Four techniques were evaluated: 1-stage bone-added osteotome sinus floor elevation procedure (BAOSFE) with simultaneous implant placement; 2-stage BAOSFE with delayed implant placement; 1-stage lateral window sinus floor elevation with simultaneous implant placement; and 2-stage lateral window sinus floor elevation with delayed implant placement. Patients were followed for 18 to 72 months (mean: 52.5 months) after prosthesis placement. Data were analyzed with cone-beam computed tomography. A total of 96 implants from 71 patients were analyzed; pretreatment, there were no significant differences between patients. Total implant survival was 98.9%. The mean residual bone height was significantly higher in the 1-stage BAOSFE group than the other groups (*P* < .01); 1 implant in this group failed at 3 months. There was no significant difference in total bone height gain between groups. However, the bone height gain of 1st sinus lifting with 2-stage BAOSFE was significantly lower than the 2-stage lateral window procedure (*P* < .01). There was no prosthesis failure. The favorable implant outcomes suggest these 1-stage and 2-stage MSFA procedures should be considered as alternative treatment options for patients with extremely atrophic posterior maxilla.

## 1 Introduction

Placement of implants in the maxillary posterior area can be a challenge for dental clinicians when there is severe atrophy. The deficiency of the residual ridge and the pneumatization of the maxillary sinus results in insufficient bone volume and poor long-term stability of the implant. This problem has been resolved with various sinus augmentation techniques to increase bone quality and quantity and protect the sinus, and the use of various implant lenghts.^[1]^

Sinus augmentation techniques include lateral window and transalveolar sinus lift with or without bone grafts. The first sinus lift procedure was performed by Tatum in 1976,^[1]^ which modified the Caldwell-Luc technique by preparing a lateral bony window to dissect and elevate the sinus membrane; following placement of autogenous bone or bone substitute in the sinus and 6 months of healing, the implant was placed. This is now referred to as a 2-stage technique: the first stage augments the sinus and implants are placed in the second stage. In 1980, Boyne and James performed a 2-stage lateral window sinus lift; implant placement was delayed for 3 months.^[1]^ In 1986, Tatum^[2]^ performed a transalveolar sinus lift, which uses a transcrestal access to the sinus from the edentulous alveolar bone; the sinus membrane is elevated by fracturing the sinus floor with vertical tapping through the alveolar ridge. Summers^[3–5]^ modified this technique in 1994 by introducing a specific set of osteotomes of different diameters to simultaneously lift the sinus floor and increase bone density. Transalveolar bone-added osteotome sinus floor elevation (BAOSFE) techniques are more conservative and result in less post-operative pain.^[6,7]^ However, there is an increased risk of complications due to the inability to visualize the Schneiderian membrane, and is considered a “blind” procedure. Although a lateral window sinus lift allows direct visualization of the Schneiderian membrane, it is more invasive; disadvantages include postoperative discomfort, complications, and an increased risk of infection.^[8,9]^

A transalveolar sinus lift is usually recommended when the initial residual alveolar bone height (RBH) is more than 5 mm; the lateral window sinus lift is suggested when the bone height is less than 5 mm.^[10]^ However, Krasny et al^[11]^ successfully reconstructed atrophic maxillary posterior ridges in 26 patients with an RBH of only 3–5 mm using a two-stage, transalveolar sinus lift technique. Krasny et al combined the benefits of low risk of complications of a transalveolar sinus lift with an extended augmentation of a lateral window sinus lift. This technique might be beneficial for patients with extremely atrophic ridges. However, no studies have compared the outcomes of this technique with those of lateral window sinus lift.

Therefore, the aim of this study was to compare implant sites from edentulous patients with a RBH of ≤3 mm treated with 4 different sinus augmentation techniques. The following techniques were compared: lateral stage sinus floor elevation, with either a 1- or 2-stage procedure, and the transalveolar BAOSFE sinus lift, with either 1- or 2-stage procedure. The following variables were used for comparison of techniques:

1. bone height gain,
2. complications
3. the influence of RBH on bone gain and implant success rate,
4. the association between techniques and implant size,
5. and length of time from the first sinus augmentation surgery to implant uncovering.

These findings could be used to determine optimal procedures for sinus augmentation when the RBH is ≤3 mm.

## 2 Materials and methods

### 2.1 Patient selection

Patients were selected by convenience sampling from a department of periodontology of a medical center in Taiwan from September, 2013 to November, 2017. All patients were candidates for this retrospective study if they had been assessed preoperatively for ridge topography and treatment planning of sinus augmentation and implant placement using cone-beam computed tomography (CBCT). Patients were included in the study if they met the following inclusion criteria:

1. partially edentulous with maxillary posterior edentulous ridge after extraction of more than 3 months;
2. ≥18 years of age;
3. initial RBH within the implantation area ≤3 mm;
4. no acute inflammation within the sinus; and
5. no systemic disease, or disease controlled with medication.

Exclusion criteria were:

1. untreated periodontitis and poor oral hygiene;
2. uncontrolled systematic disease;
3. treated or under treatment with bisphosphonates;
4. previous irradiation in the head and neck area;
5. pregnant;
6. contraindications to implant surgery;
7. poor motivation;
8. sinus membrane perforation; or
9. current smoker.

### 2.2 Group assignments

A total of 71 patients met the inclusion criteria and were placed into groups based on the sinus lifting technique used for implant placement: 1-stage BAOSFE, simultaneous implant placement (B-1); 2-stage BAOSFE, delayed implant placement (B-2); 1-stage lateral window sinus lift, simultaneous implant placement (L-1); and 2-stage lateral window sinus lift, delayed implant placement (L-2).

### 2.3 Sinus lift techniques

All surgical procedures were performed under local anesthesia. First, a subcrestal incision was made near the palatal side, extending to more than one tooth mesially, and a full-thickness mucoperiosteal flap was elevated. Simultaneous or delayed implant placement was performed depending on sinus lift technique (Boyne and James, 1980;^[1]^ and Summers, 1994^[3–5]^). All procedures used xenograft (Bio-Oss, Geistlich, Switzerland) for bone grafts and Biomet 3i implants (USA).

**Figure 1:**
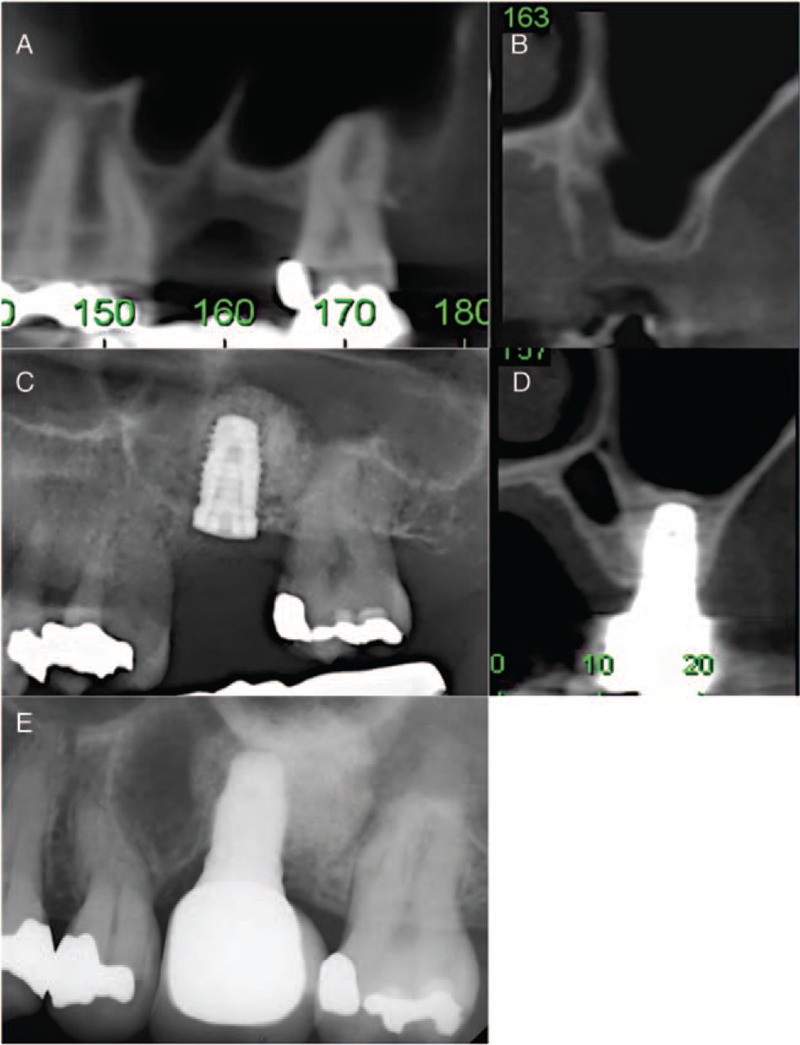
Representative CBCT radiographs demonstrate procedural steps for 1-stage bone-added osteotome sinus floor elevation (BAOSFE) (B-1 group). (A, B) Pre-operative radiographs show limited residual bone height (RBH) over the upper left first molar region (RBH: 2.3 mm). (C, D) Radiographs obtained after BAOSFE and simultaneous implant placement. (E) Radiograph obtained 18 months after prosthesis delivery.

**Figure 2:**
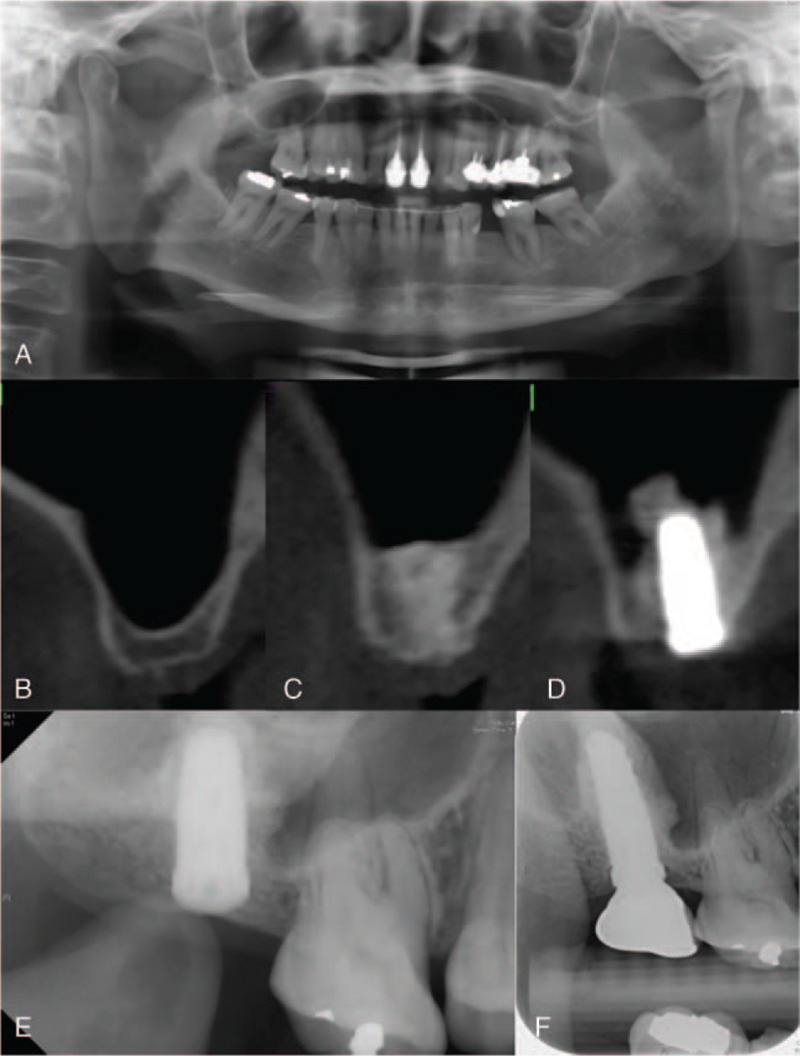
Representative CBCT radiographs demonstrate procedural steps for 2-stage bone-added osteotome sinus floor elevation procedure (BAOSFE) (B-2 group). (A, B) Pre-operative radiographs show extremely atrophic residual bone height (RBH) over the upper right second molar region (RBH: 2.7 mm). (C) Radiograph obtained immediately after first stage of 2-stage BAOSFE. (D, E) Radiographs obtained 6 months after sinus lift, immediately following implant placement (2nd stage). (F) Radiograph obtained 18 months after prosthesis delivery.

The 1-stage and 2-stage BAOSFE (B-1 and B-2, respectively) was performed with xenograft to elevate the sinus membrane to at least 10 mm. Simultaneous implant placement and suturing for primary closure was performed in the B-1 procedure (Fig. 1A–C). A period of at least 6 months was allowed for graft healing, at which time implant osseointegration was assessed; implant uncovering and prosthesis fabrication were performed sequentially (Fig. 1D–E). For the B-2 procedure, implants were placed 6 months or more after sinus augmentation (Fig. 2A–F). If the ridge height was determined to be insufficient at this time, BAOSFE was performed again before implant placement.

**Figure 3:**
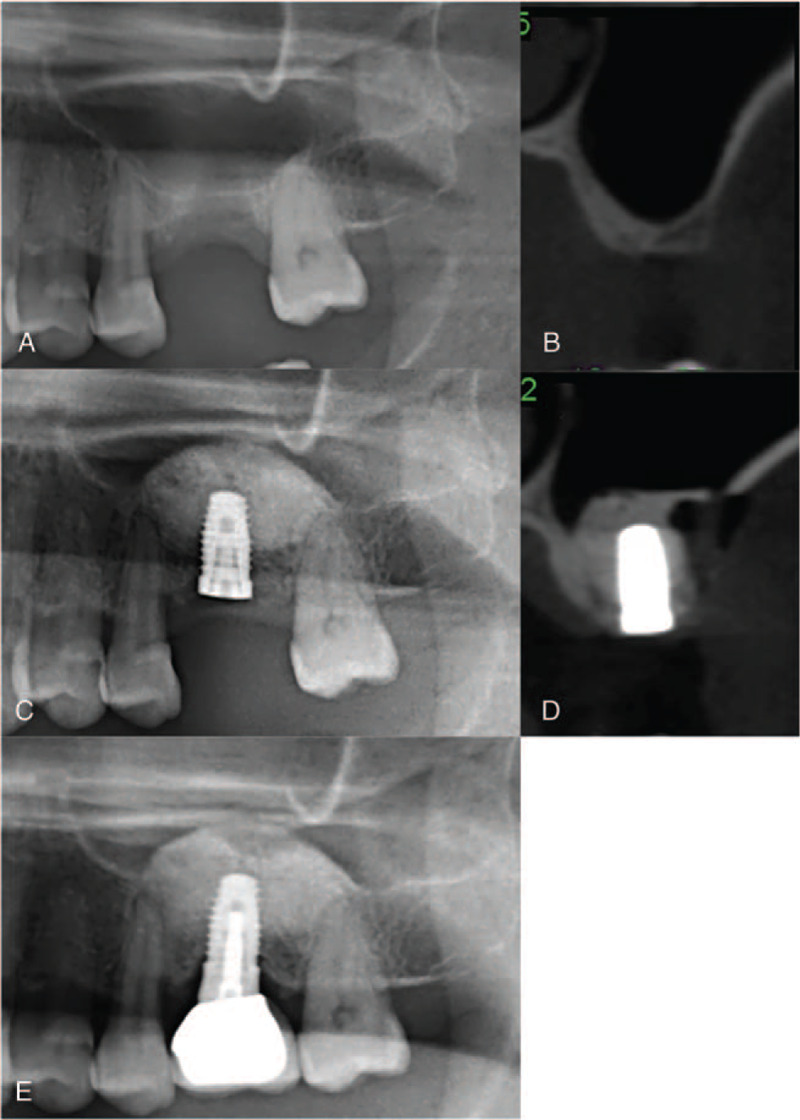
Representative CBCT radiographs demonstrate procedural steps for the 1-stage lateral window sinus lift (L-1 group). (A, B) Pre-operative radiographs show extremely atrophic residual bone height (RBH) at upper left first molar region (RBH: 2 mm). (C, D) Radiographs obtained immediately after lateral window sinus lift with simultaneous implant placement. (E) Radiograph obtained 18 months after prosthesis delivery.

**Figure 4:**
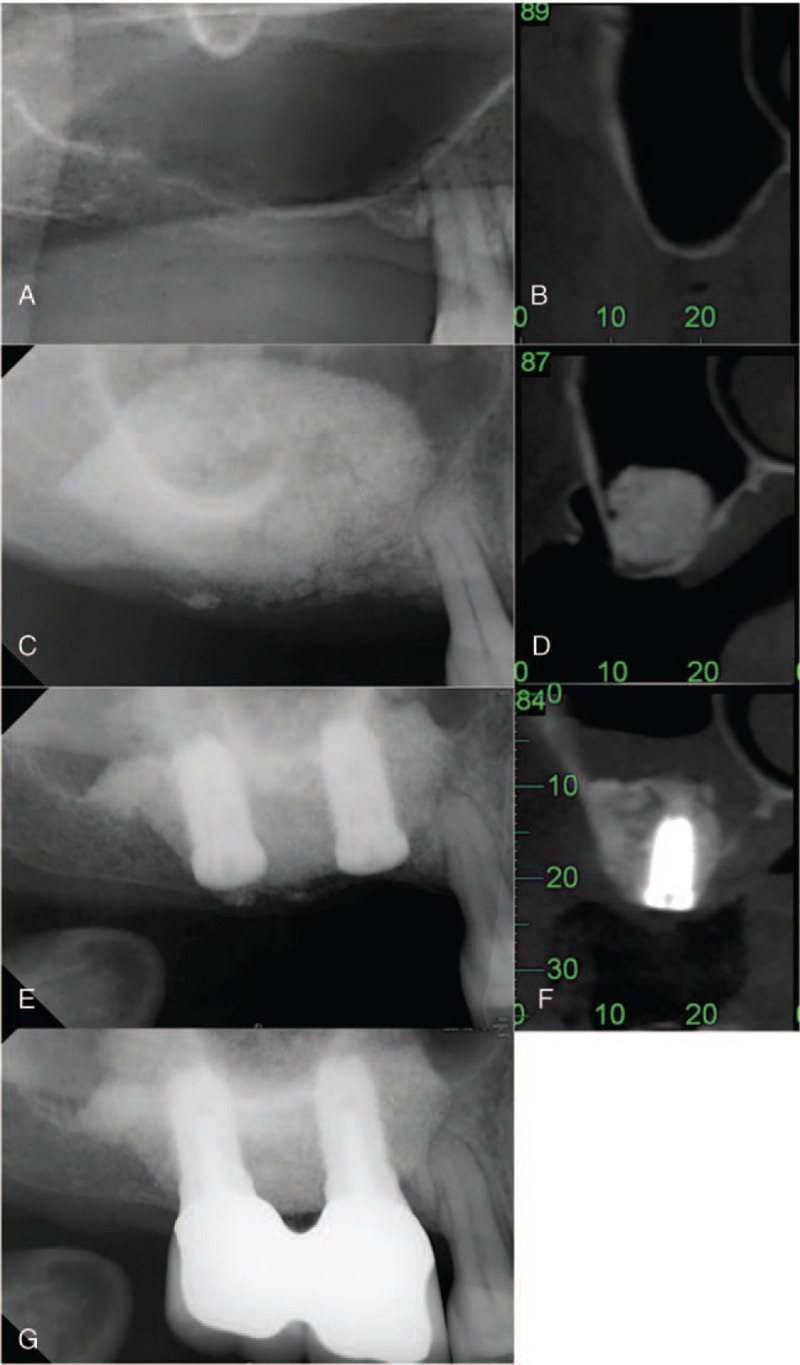
Representative CBCT radiographs demonstrate procedural steps for the 2-stage lateral window sinus lift (L-2 group). (A, B) Pre-operative radiographs show extremely atrophic residual bone height (RBH) over the right upper molar region (RBH: 1.5∼2 mm). (C, D) Radiographs obtained immediately after first stage of 2-stage lateral window sinus lift. (E, F) Radiographs obtained 6 months after lateral window sinus lift, immediately following implant placement (2nd stage). (G) Radiograph obtained 18 months after prosthesis delivery.

The 1- and 2-stage lateral window sinus lifting (L-1 and L-2, respectively) was performed by preparing a lateral bony window with round-headed diamond burs; the sinus membrane was elevated by excavators. For the L-1 procedure, the inner part of the sinus cavity was grafted with xenograft, followed by simultaneous implant placement (Fig. 3A–D). The graft was then packed around and over the implants. A collagen membrane (Osseoguard, Collagen Matrix, Inc, USA) covered the lateral bony window and primary closure by suturing was performed. A graft healing period of at least 6 months was followed by assessment of implant osseointegration, and sequential implant uncovering and prosthesis fabrication were performed (Fig. 3e). For the L-2 procedure, a graft healing period of more than 6 months occurred following sinus lift prior to implant placement (Fig. 4A–G). If the ridge height was determined to be insufficient at this time, transalveolar sinus lift was performed before the implant placement.

### 2.4 Measurements

**Figure 5:**
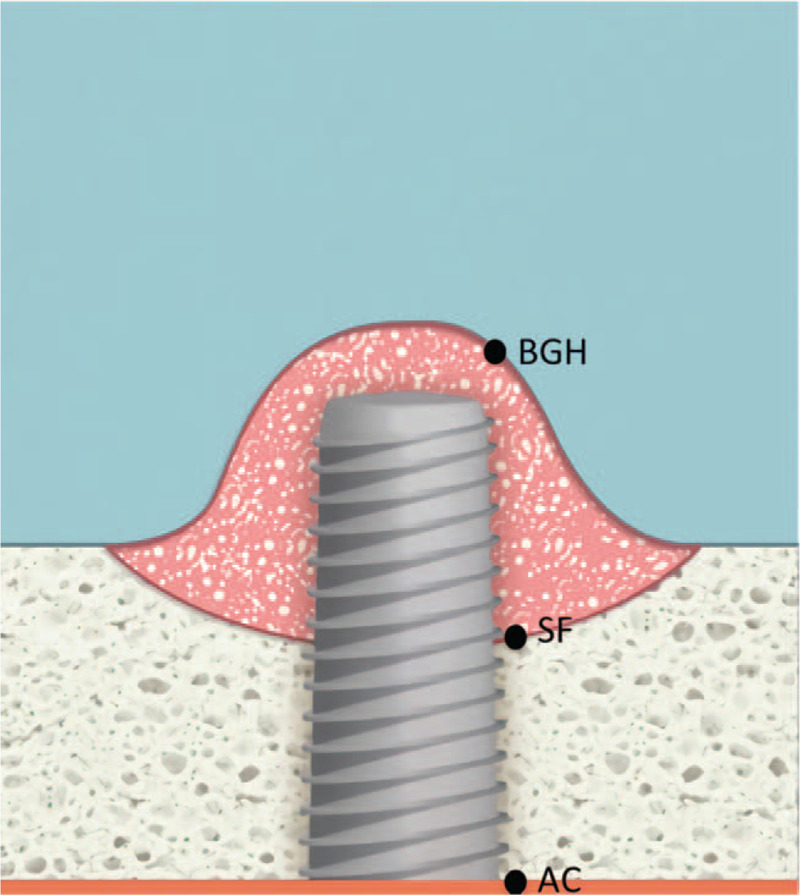
Schematic showing areas measured with cone-beam computed tomography. AC = alveolar crest, AC-SF = pre-operative residual bone height (RBH), BGH = bone graft height; SF = sinus floor, SF-BGH = bone height gain in the sinus.

Measurements were determined by clinical examination and CBCT radiographs. Data were collected for each group regarding implants per patient, position of implant, and implant length and width. CBCT images were used for measures of RBH before surgery and total bone height gain after all treatments were complete for 4 groups. Graft healing time and the bone height gain of 1st sinus lifting prior to implant placement was determined for both 2-stage surgery groups (B-2 and L-2). Figure 5 shows a schematic of these areas.

### 2.5 Statistical analysis

Analyses were performed using the statistical software package SPSS (Version 17.0.1, Armonk, NY, USA) and Excel (Microsoft, Seattle, WA, USA). Descriptive statistics of implant characteristics for the 4 treatment groups were calculated as means and standard deviations (SD). The distribution of the implant length and width for each group was analyzed by Chi-Squared test. The Mann–Whitney *U* test was used to compare differences in graft healing time and the bone height gain of 1st sinus lifting between B-2 and L-2 groups after the first stage of surgery. The Kruskal–Wallis test evaluated differences between the 4 groups for RBH, total bone height gain, and surgical treatment period. The significance level for all statistical tests was set at *P* < .05.

## 3 Results

A total 96 implants were placed in 71 patients. Patient characteristics are shown in Table 1. The mean age of the patients did not differ significantly between groups. Number of implants between groups differed significantly (*P* < .01). In groups B-1, B-2, and L-2, most patients had one implant placement: 84.2% in B-1, 73.9% in B-2, and 64.7% in L-1. However, 66.7% of patients (n = 8) in group L-2 received 2 implants, and this was the only group in which 1 patient received 4 implants. The mean number of days of surgical treatment (from first surgery to completion of the last surgery) differed between groups (*P* < .01); for the B-1 and L-1 groups the mean was 270.27 days (standard deviation, SD = 114.99) and 263.00 days (SD = 63.02), respectively; compared with 401.43 days (SD = 117.32) and 458.87 days (SD = 139.98) for B-2 and L-2 groups, respectively.

**Table 1:** Characteristics of patients (N = 71) in each implant treatment group.

	Implant treatment group				
Characteristic	B-1 (n = 19)	B-2 (n = 23)	L-1 (n = 17)	L-2 (n = 12)	*P*
Age, years (mean ± SD)	55.91 ± 5.06	51.86 ± 13.46	53.52 ± 10.97	55.74 ± 6.60	.813^†^
Implants per patient, n (%)					<.01^§^
1	16 (84.2)	17 (73.9)	11 (64.7)	3 (25)	
2	3 (15.8)	5 (21.7)	6 (35.3)	8 (66.7)	
3	0 (0.0)	1 (4.4)	0 (0.0)	0 (0.0)	
4	0 (0.0)	0 (0.0)	0 (0.0)	1 (8.3)	
Total treatment, days (mean ± SD)	270.27 ± 114.99	401.43 ± 117.32	263.00 ± 63.02	458.87 ± 139.98	<.01^†^

Implant positions and sizes for treatment groups are shown in Table 2. Implant position did not differ significantly between groups; most were in the molar position; ranging from 77.3% (B-1) to 95.7% (L-2) of implants. There was a significant difference between groups in length of implant used (*P* < .01); 63.4% in the B-1 group were 8.5 mm, whereas the B-2 and L-1 group used more 11.5 mm implants (57.1% and 47.8%, respectively). Most implants in the L-2 group (56.5%) were 10 mm. There was no significant difference in implant width between groups.

**Table 2:** Characteristics of implants and implant sites (N = 96) in the 4 treatment groups.

	Implant treatment group				
Characteristic	B-1 (n = 22)	B-2 (n = 28)	L-1 (n = 23)	L-2 (n = 23)	*P*
Position of implant, n (%)					.29^§^
Pre-molar	5 (22.7)	4 (13.8)	5 (21.7)	1 (4.3)	
Molar	17 (77.3)	24 (85.7)	18 (78.3)	22 (95.7)	
Implant length, n (%)					<.01^§^
8.5 mm	14 (63.4)	3 (10.7)	1 (4.3)	0 (0.0)	
10.0 mm	7 (31.8)	9 (32.1)	8 (34.7)	13 (56.5)	
11.5 mm	1 (4.5)	16 (57.1)	11 (47.8)	9 (39.1)	
13.0 mm	0 (0.0)	0 (0.0)	3 (13.0)	1 (4.3)	
Implant width, n (%)					.586^§^
3.25 mm	2 (9.1)	2 (7.1)	1 (4.3)	0 (0)	
4.00 mm	3 (13.6)	8 (28.6)	8 (34.7)	7 (30.4)	
5.00 mm	17 (77.3)	18 (64.2)	14 (60.1)	16 (69.6)	
Measurements, Mean ± SD					
RBH, pre-treatment, mm	2.78 ± 0.45^∗∗^	2.16 ± 0.73	2.27 ± 1.14	1.28 ± 0.77	<.01^†^
Graft healing time, months		7.59 ± 1.99		9.73 ± 2.11	.069^‡^
Bone height gain of 1st sinus lifting, mm		5.43 ± 2.21		8.44 ± 2.72^∗∗^	<.01^‡^
Total bone height gain, mm	8.31 ± 1.10^∗∗^	10.83 ± 1.59	11.55 ± 1.56	12.85 ± 2.01	<.01^†^

Pre- and post-treatment measures of implant sites were determined from CBCT images (Table 2). All implants had an initial RBH ≤3 mm. The mean RBH was 2.78 mm (SD = 0.45) for the B-1 group, significantly higher than the other groups (*P* < .01) (compare Fig. 1 (A, B) to Figs. 2–4 (A, B). There was no significant difference in graft healing time between B-2 and L-2 groups, which was at least 6 months. However, mean bone height gain of 1st sinus lifting at time of implant for the L-2 group (8.44 mm, SD = 2.72) was significantly greater than the B-2 group (5.43 mm, SD = 2.21) (compare Fig. 2 (D, E) to Fig. 4 (D, E). Mean total bone height gain, determined for all groups when treatment was complete, was 8.31 mm (SD = 1.10) for the B-1 group, which was significantly less (*P* < .01) than for the B-1, L-1, and L-2 groups (10.83 (SD = 1.59), 11.55 (SD = 1.56), and 12.85 (SD = 2.01), respectively).

Outcome measures 18 to 72 months (mean: 52.5 months) following prosthesis placement did not differ significantly between groups; there was no prothesis failure. Figs. 1E, 2F, 3E, and 4G show radiographs of prostheses 18 months following delivery for B-1, B-2, L-1, and L-2 treatment, respectively. There was only one implant failure, which occurred in the molar region in a B-1 patient. The implant was removed 6 months following placement; implant size was 5 × 10 mm, and RBH was 3 mm. After a 3-month healing period, a new implant was placed and the prosthesis was delivered after 10 months; the implant survived more than 4 years.

## 4 Discussion

This is the first study to evaluate 4 different techniques of sinus augmentation for implant placement in patients with an initial RBH of ≤3 mm. Assessment of implants 18 to 72 months (mean: 52.5 months) following placement of prostheses showed comparable outcomes for all sinus lift techniques; nearly all implants (95 of 96) survived for the entire follow-up period (18–72 months, mean: 52.5 months). Treatment time was significantly longer for implant placement requiring 2-stages (B-2 and L-2 groups) than for 1-stage (B-1 and L-1 groups), as would be expected. However, treatment time did not differ between the 1-stage or 2-stage groups.

Previous studies have suggested sinus augmentation with BAOSFE should be limited to patients with an RBH of ≥5 mm; lateral window sinus lift should be performed when the RBH is ≤4 mm_._^[3–5,12]^ Rosen et al^[13]^ found success of implant placement using BAOSFE was better when the RBH was ≥ 5 mm, regardless of whether a 1-stage or 2-stage procedure was used. A meta-regression analysis of the association between RBH and success of implants following lateral window or osteotome sinus elevation techniques by Chao et al^[14]^ found implant survival rates with a lateral window sinus lift were positively associated when the RBH was ≥5 mm. However, no relationship could be determined for transalveolar sinus lift techniques because the included studies lacked sufficient data for an initial RBH of ≤4 mm. A more recent meta-analysis by Calin et al^[15]^ showed an initial RBH of >4 mm did not impact implant success or failure; however, an initial RBH of <4 mm was positivity associated with implants inserted in combination with transalveolar sinus elevation techniques. Our findings demonstrated high implant success rates with an initial RBH of ≤3 mm for both lateral window and transalveolar techniques, which suggests the influence of RBH on the success of different sinus augmentation procedures deserves further in-depth assessment to determine what variables might influence outcomes. For instance, the high technical ability required for successful osteotome sinus elevation techniques^[14]^ or inclusion/exclusion criteria for selection of patients could contribute to variations in outcomes.

A lateral window sinus lift technique has been shown to produce a greater bone height gain without the limitation of the size of the pre-operative RBH.^[6,16]^ Our success with implant placement in the L-1 and L-2 group is further evidence that RBH is not a limitation for lateral window sinus lift; total bone height gain was similar for both groups. Both transalveolar sinus lift procedures also resulted in long-term survival of implants. The mean total bone height gain of 8.31 mm for the B-1 group is similar to a study by Winter et al.^[17]^ They found the mean bone height gain for implants (n = 58) placed with a 1-stage transalveolar sinus lift and an initial RBH of <4 mm was 9.12 mm. Mean bone height gain of 1st sinus lifting in the B-2 group was 5.43 mm, which is greater than the gain of 3.94 mm for 26 implants reported by Krasny et al;^[11]^ however, the mean RBH in their study was 4.22 mm. There were significant differences in the bone height gain of 1st sinus lifting for the 2-stage surgery groups (B-2 and L-2) following graft healing; the mean gain in the B-2 group (5.43 mm, SD = 2.21) was significantly lower than the L-2 group (8.44 mm, SD = 2.72). However, the total bone height gain when all treatments were complete was not significant between the B-2 and L-2 group. Our findings revealed that the 2nd BAOSFE could compensate for the 1st BAOSFE in 2-stage surgery group, increase total bone height and achieve the comparable treatment outcomes to the lateral window sinus lifting.

In the present study, the implants were all >8 mm in length, ranging from 8.5 to 13 mm, and all but one were successful. These findings are in contrast to those reporting an association of shorter implants with lower success rates. In earlier studies, short implants were defined as an infrabony length of less than 8 mm.^[18]^ Whereas the predictability of standard implants ≥10 mm is high because a longer length provides better distribution of functional forces throughout the implants.^[19,20]^ More recent studies have reported comparable survival rates for short and standard-length implants. For instance, a systemic review of 17 studies using short implants (<8 mm) with observation periods ranging from 3 months to 9 years found survival rates ranged from 92.2% to 100%.^[21]^ A systemic review of 33 studies found no significant difference in survival rates between short and long implants.^[22]^ However, Thoma et al^[23]^ reviewed 5 randomized controlled clinical trials with 16 to 18 months follow-up and compared short implants (≤8 mm) in the posterior maxilla to longer implants (>8 mm) placed after or simultaneously with the transalveolar or lateral window sinus elevation procedures. Survival rates were similar for both longer and shorter implants (99.5% and 99.0%, respectively). However, complications were higher (almost 3 times) for longer implants in the augmented sinus, mainly due to membrane perforations during surgeries. There were no membrane perforations for any of the implants in our study regardless of implant length. The one implant that failed was 10 mm in length; the lack of infection suggests the loss was most likely due to a failure to achieve osseointegration. Our findings suggest a pre-operative size of ≤3 mm for RBH is not necessarily predictive of implant failure, but may important for determining how much bone gain can be achieved. Therefore, the initial RBH should be a component when considering implant length relative to the amount of bone gain needed.

The advantages of the 1-stage sinus lift techniques are fewer surgical procedures and less healing time; however, simultaneous placement may prevent the implant from achieving primary stability and may increase the risk of failure. The 50% shorter healing time with the 1-stage sinus lift and implant surgery has been previously reported.^[24]^ Although the 2-stage procedures had significantly longer treatments periods than the 1-stage procedures for both the B-2 and L-2 groups, the only implant to fail was in the B-1 group, and this occurred prior to the implant uncovering procedure.

This study had some limitations. First, this was a retrospective study; a prospective randomized clinical study should be conducted in order to confirm our findings. Second, the sample size in each group was small. Future studies with more patients and a longer follow-up period could strengthen these findings.

In summary, comparable and desirable outcomes were achieved for all patients with an RBH ≤3 mm, regardless of implant placement technique. The strength of these findings lies in the broad representation of implant size and width across 4 different sinus lift procedures. Although there were significant differences in surgical treatment times, and the initial RBH between the 4 groups, and the bone height gain of 1st sinus lifting between the 2-stage surgery groups, there was no difference in implant success 18 to 72 months (mean: 52.5 months) following prosthesis delivery. BAOSFE with staged procedures is not only less invasive than the lateral window sinus lift technique, but predictability is greater than with one-stage procedures, which may make it more acceptable for patients. These findings provide dental clinicians with evidence that alternative options should be considered for sinus augmentation surgery when patients present with extremely atrophic posterior maxilla. However, the more complex technical ability required for successful osteotome sinus elevation techniques will require careful preoperative planning and meticulous surgical skills.

## Acknowledgments

The authors would like to thank Dr. Pan YC, Dr. Kung CY, Dr. Hsu YH and Dr. Kuo PY for guidance and enthusiastic encouragement.

## Author contributions

**Data curation:** Whei-Lin Pan, Yi-Ping Pan, Chia-Fang Tsai.

**Resources:** Whei-Lin Pan, Chiu-Po Chan.

**Formal analysis:** Yi-Ping Pan, Chia-Fang Tsai.

**Supervision:** Whei-Lin Pan.

**Writing – original draft:** Chia-Fang Tsai.

**Writing – review & editing:** Whei-Lin Pan, Yi-Ping Pan, Chiu-Po Chan, Yuh-Ren Ju, Yuan-Min Wang, Cho-Ying Lin, Chi-Ching Chang.
